# Supplementary material for: First birth and the trajectory of women’s empowerment in Egypt
Source: BMC Pregnancy Childbirth. 2017 Nov 8;17(Suppl 2):362. doi: 10.1186/s12884-017-1494-2 (PMC5688501; doi:10.1186/s12884-017-1494-2)
Supplement: Supplementary file 1 — (DOCX 15 kb) [file 12884_2017_1494_MOESM1_ESM.docx]

| **Appendix Table 1. Fertility and Sample Characteristics (Means (SD) or %) of Lost to Follow Up Married Women Ages 15 to 49, Egyptian Labor Market Panel Survey** | | |
| --- | --- | --- |
|  | **Lost to Follow Up  Married Women N=1,181** | |
| Key Variables in 2006 | N | % or Mean (SD) |
| **Number of Births** |  |  |
| **0** | 201 | 17.02 |
| **1** | 324 | 27.43 |
| **2** | 288 | 24.39 |
| **3** | 191 | 16.17 |
| **4+** | 177 | 14.99 |
| Mean (SD) | 1,181 | 2.00 (1.69) |
| **Gender of Firth Birth** |  |  |
| Boy | 467 | 47.7 |
| Girl | 512 | 52.3 |
| **Current Age (years)** | 1,181 | 31.0 (8.29) |
| 15 - 24 years | 297 | 25.15 |
| 25 - 34 years | 500 | 42.34 |
| 35 - 44 years | 278 | 23.54 |
| 45 - 49 years | 106 | 8.98 |
| **Years of Education** | 1,181 | 9.54 (5.54) |
| **Age at Marriage (years)** | 1,181 | 22.2 (4.41) |
| Less than 18 years old | 173 | 14.7 |
| 18 years or older | 1,008 | 85.4 |
| **Value of Dowry** |  |  |
| No Response | 291 | 24.64 |
| No Amount | 558 | 47.25 |
| Some Amount | 332 | 28.11 |
| **Related to Husband (1/0)** | 1,181 | 22.4 |
| **Ever Worked (1/0)** | 1,181 | 31.4 |
| **Region** |  |  |
| Greater Cairo | 374 | 31.67 |
| Alexandria & Suez Canal | 217 | 18.37 |
| Urban Lower | 174 | 14.73 |
| Urban Upper | 132 | 11.18 |
| Rural Lower | 152 | 12.87 |
| Rural Upper | 132 | 11.18 |
| **Household Wealth Index** |  |  |
| Poorest | 91 | 7.71 |
| Poorer | 178 | 15.07 |
| Middle | 236 | 19.98 |
| Richer | 330 | 27.94 |
| Richest | 346 | 29.3 |
| **Husband's Age in years** | 1,181 | 37.5 (9.93) |
| **Husband's Years of Education** | 1,181 | 10.5 (5.32) |
|  |  |  |

| **Appendix Table 2. Negative Binomial Multilevel Regression Models of Combined Decision-Making in 2012, Egyptian Labor Market Panel Survey (N=4,660)** | | |
| --- | --- | --- |
|  | **Combined Decision-Making 2012** | |
|  | Negative Binomial | |
| **Key Variables** | IRR | (SE) |
| **Births 2006** (Ref=1 Birth) |  |  |
| **0** | 0.85*** | (0.04) |
| **2** | 1.24*** | (0.04) |
| **3** | 1.42*** | (0.06) |
| **4+** | 1.53*** | (0.07) |
| **Combined Household Decision Making 2006** | 1.03*** | (0.00) |
| **Mobility 2006** | 1.10*** | (0.02) |
| **Financial Autonomy 2006** | 1.06* | (0.03) |
| Age (years) | 0.94*** | (0.00) |
| Years of Education | 1.02*** | (0.00) |
| Less than 18 years old at marriage | 0.87*** | (0.03) |
| Dowry (Ref=None) |  |  |
| No Response | 0.97 | (0.03) |
| Some | 0.99 | (0.03) |
| Related to Husband | 0.99 | (0.02) |
| Ever Worked | 1.09** | (0.03) |
| Region (Ref=Greater Cairo) |  |  |
| Alexandria & Suez Canal | 0.96 | (0.06) |
| Urban Lower | 0.96 | (0.05) |
| Urban Upper | 0.86** | (0.04) |
| Rural Lower | 0.96 | (0.05) |
| Rural Upper | 0.77*** | (0.04) |
| Household Wealth Index (Ref=Poorest) |  |  |
| Poorer | 0.94 | (0.03) |
| Middle | 0.93* | (0.03) |
| Richer | 0.95 | (0.04) |
| Richest | 0.89* | (0.04) |
| Husband's Age (years) | 1.00 | (0.00) |
| Husband's Years of Education | 1.00 | (0.00) |
| **Variance at Level 1 (Individual Level)** |  |  |
| **Variance at Level 2 (PSU Level)** | 0.55 | (0.09) |
| Notes: *p<0.05, ** p<0.01, *** p<0.001. Standard errors in parentheses | | |
